# DNA methylation signature of human hippocampus in Alzheimer’s disease is linked to neurogenesis

**DOI:** 10.1186/s13148-019-0672-7

**Published:** 2019-06-19

**Authors:** Miren Altuna, Amaya Urdánoz-Casado, Javier Sánchez-Ruiz de Gordoa, María V. Zelaya, Alberto Labarga, Julie M. J. Lepesant, Miren Roldán, Idoia Blanco-Luquin, Álvaro Perdones, Rosa Larumbe, Ivonne Jericó, Carmen Echavarri, Iván Méndez-López, Luisa Di Stefano, Maite Mendioroz

**Affiliations:** 1 Neuroepigenetics Laboratory, Navarrabiomed, Public University of Navarre (UPNA), IdiSNA (Navarra Institute for Health Research), c/ Irunlarrea, 3, 31008 Pamplona, Spain; 2 Department of Neurology, Complejo Hospitalario de Navarra, IdiSNA (Navarra Institute for Health Research), Pamplona, Spain; 3 Department of Pathology, Complejo Hospitalario de Navarra- IdiSNA (Navarra Institute for Health Research), Pamplona, Spain; 4 Bioinformatics Unit, Navarrabiomed, Public University of Navarre (UPNA), IdiSNA (Navarra Institute for Health Research), Pamplona, Spain; 5 Laboratoire de biologie cellulaire et moléculaire du contrôle de la prolifération (LBCMCP), Université Paul Sabatier, CNRS, Toulouse, France; 6 Department of Internal Medicine, Hospital García-Orcoyen, Estella, Spain

**Keywords:** DNA methylation, Alzheimer’s, Hippocampus, Adult neurogenesis, Poised promoters, Homeobox, Neurodevelopment, Epigenetics

## Abstract

**Background:**

Drawing the epigenome landscape of Alzheimer’s disease (AD) still remains a challenge. To characterize the epigenetic molecular basis of the human hippocampus in AD, we profiled genome-wide DNA methylation levels in hippocampal samples from a cohort of pure AD patients and controls by using the Illumina 450K methylation arrays.

**Results:**

Up to 118 AD-related differentially methylated positions (DMPs) were identified in the AD hippocampus, and extended mapping of specific regions was obtained by bisulfite cloning sequencing. AD-related DMPs were significantly correlated with phosphorylated tau burden. Functional analysis highlighted that AD-related DMPs were enriched in poised promoters that were not generally maintained in committed neural progenitor cells, as shown by ChiP-qPCR experiments. Interestingly, AD-related DMPs preferentially involved neurodevelopmental and neurogenesis-related genes. Finally, InterPro ontology analysis revealed enrichment in homeobox-containing transcription factors in the set of AD-related DMPs.

**Conclusions:**

These results suggest that altered DNA methylation in the AD hippocampus occurs at specific regulatory regions crucial for neural differentiation supporting the notion that adult hippocampal neurogenesis may play a role in AD through epigenetic mechanisms.

**Graphical abstract:**

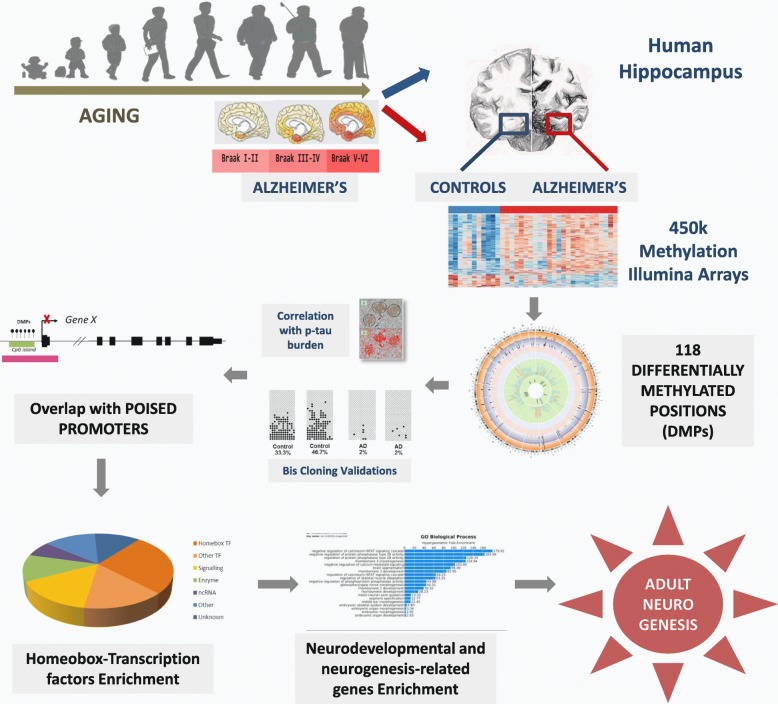

**Electronic supplementary material:**

The online version of this article (10.1186/s13148-019-0672-7) contains supplementary material, which is available to authorized users.

## Background

Alzheimer’s disease (AD) is the leading cause of age-related dementia and one of the major global challenges of our time [1]. As knowledge about AD increases, so does the appreciation of the pathogenic complexity of the disorder [2]. Currently, AD is considered a complex disease that arises from the interaction between environmental and genetic factors [3], modulated through epigenetic mechanisms. Since epigenetics acts as an interface between the environment and the genome, a major focus has been upon studying epigenetic alterations in AD to shed some light on the pathogenesis of the disease. DNA methylation is a major epigenetic modification that involves the attachment of a methyl group to the 5-carbon position of a cytosine residue and usually occurs at cytosine-guanine dinucleotides (CpG). These CpG dinucleotides are clustered in the genome constituting CpG islands, which are enriched in the promoter of more than half of human genes and other important regulatory regions.

DNA methylation is known to be altered in complex diseases including AD. Indeed, a number of gene-specific differences in DNA methylation have been reported so far [4–8]. More recently, genome-wide approaches have uncovered additional gene-specific methylation differences across different brain regions in AD [9, 10]. By using Illumina Infinium HumanMethylation450K arrays, several genes have been found to be differentially methylated in AD brain autopsy samples, including some genes previously identified as harboring genetic variants for AD, such as *ANK1* (ankyrin-1) or *BIN1* (amphiphysin II) [11–14]. Importantly, a number of these DNA methylation marks are present in early stages of AD, suggesting that such changes might play a role in the onset of the disorder [12]. On the whole, these reports are providing significant data to enrich our understanding of AD pathogenesis [15, 16].

So far, genome-wide DNA methylation studies on AD have been performed across different brain regions including prefrontal, frontal, and superior temporal neocortex, along with entorhinal cortex. In this study, we have taken a complementary strategy to profile genome-wide DNA methylation in the human hippocampus, a brain region particularly vulnerable to AD [17–19] and the core of pathological protein tau deposits [20]. We have applied Infinium HumanMethylation450 BeadChip array to hippocampal samples obtained from a homogeneous cohort of pure AD brains and controls. As a result, we report on novel gene-specific DNA methylation changes, recurrent across multiple affected subjects, which occur in the AD hippocampus. This DNA methylation signature of the AD hippocampus correlates with tau burden and also with specific changes in histone marks. Finally, in silico functional analysis of these changes points to molecular and biological alterations that may be especially relevant to the pathogenesis of AD, including adult brain neurogenesis.

## Results

### Hippocampal samples from AD patients and controls

DNA methylation changes were evaluated in 36 postmortem hippocampal samples obtained from 26 patients with AD and 12 control subjects. To avoid spurious molecular findings related to multiprotein deposits, only AD cases with pure deposits of p-tau and β-amyloid were eligible for the study and controls were free of any protein aggregates. This approach maximizes the chances of finding true molecular associations with AD, even though reducing the number of older controls.

Neuropathological and demographic features of subjects, including age, gender, ABC score, and postmortem interval (PMI), are listed in Additional file 1: Table S1. AD subjects were older than controls (81.2 ± 12.1 versus 50.7 ± 21.5; *p* value< 0.01), and no differences were found regarding gender (*p* value = 0.16). The PMI ranged from 1.4 to 33 h and were not significantly different between groups (8.2 ± 4.2 h in controls versus 7.9 ± 7.1 h in AD samples; *p* value = 0.91).

Since DNA methylation may be affected by cellular composition of the brain samples, cell proportions were estimated by using the CETS R package as previously described [14, 21]. No statistically significant differences were found in cell proportions between the AD group and the control group (control mean neuronal cell proportion = 0.18 versus AD mean neuronal cell proportion = 0.20, *p* value = 0.64).

### Characterization of differentially methylated positions in the AD hippocampus

**Fig. 1:**
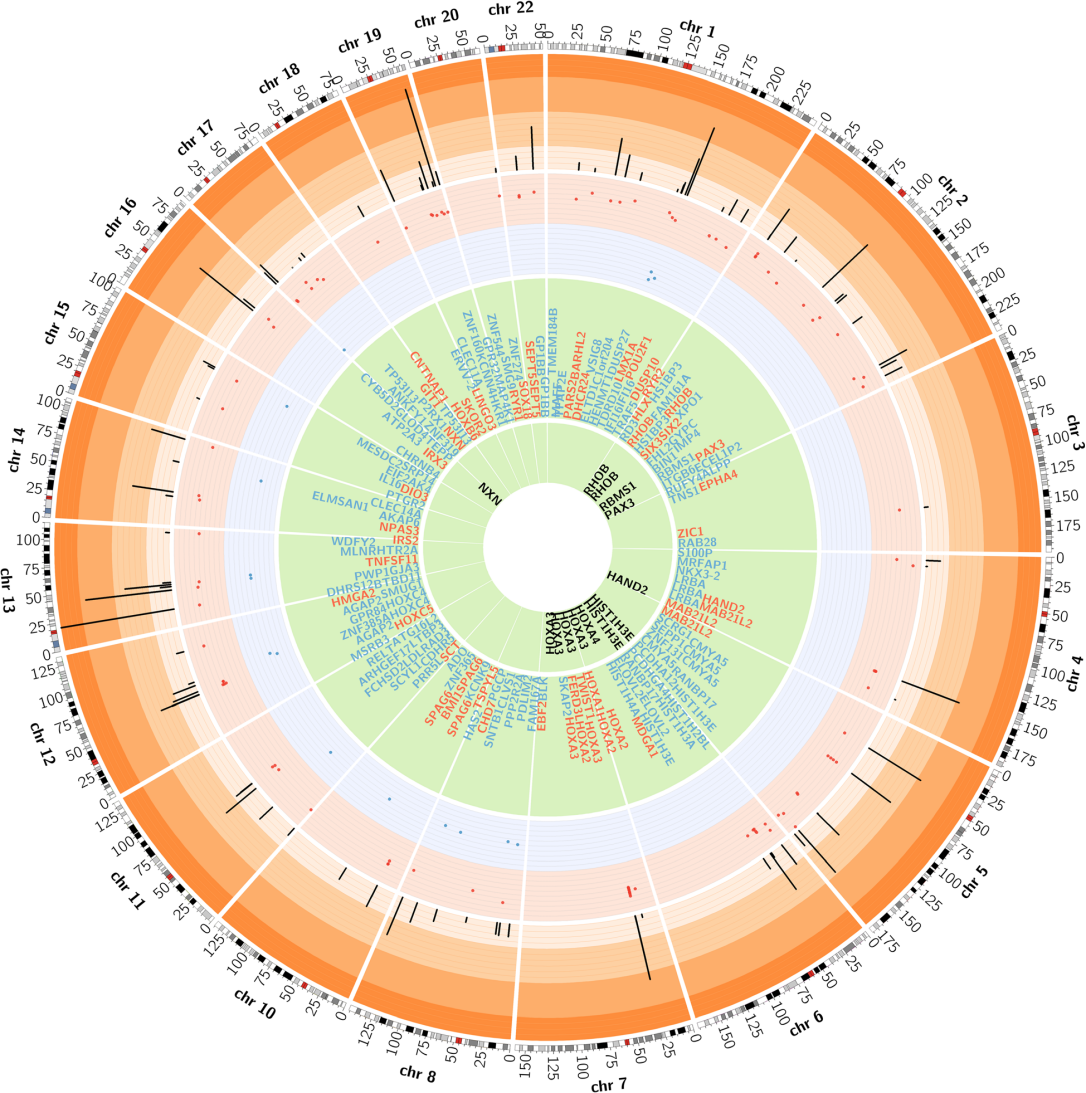
CIRCOS plot of differentially methylated positions (DMPs) in AD hippocampus. The CIRCOS plot shows a summary of DNA methylation screening results in the AD hippocampus and its validation by bisulfite cloning sequencing. The perimeter of the circular figure represents the human chromosomes, showing the cytogenetic bands and centromeres (in red). Only those chromosomes harboring DMPs are represented in the painted circles. X and Y chromosomes were excluded from the analysis. The orange circle represents *p* value for each DMP. The inner red and blue dots represent the results of the differential analysis (beta difference) for each DMPs, including gains in methylation (red dots) and losses in methylation (blue dots). The next green circle reports the names of the genes associated to each DMPs. Those genes associated with neurogenesis are highlighted in red font. In black font, those genes that were validated by bisulfite cloning sequencing are shown

**Fig. 2:**
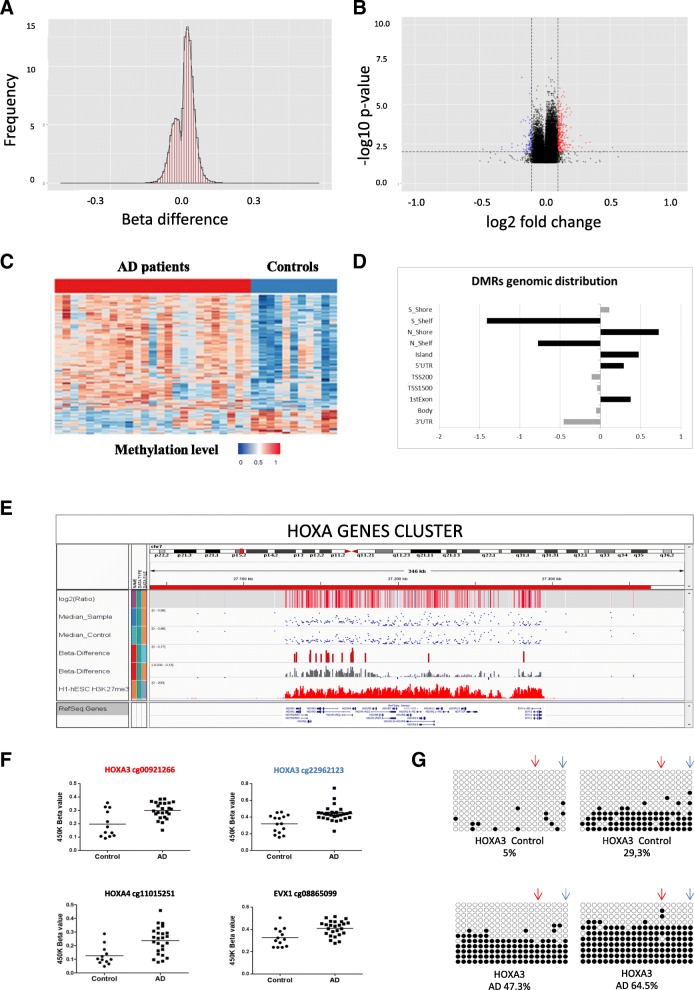
Characterization of AD-related DMPs. **a** The histogram of beta difference value distribution per CpGs shows a clear bias toward the hypermethylated changes (> 0.0) in AD hippocampal samples compared to controls. **b** The volcano plot shows a greater number of hypermethylated marks (red dots) compared to hypomethylated marks (blue dots) that crossed the statistical thresholds (dotted lines) in this study. The graph also shows that DNA methylation changes in AD hippocampus are mild to moderate in effect size. **c** The heat map graph reveals that most of the DNA methylation changes represent gains (red squares) in methylation. **d** Distribution of DMPs regarding gene structure. The bar graph shows the log2 ratios of observed (fraction of differentially methylated probes that overlap a particular region) to expected (fraction of probes selected for analysis that overlap a particular region). S = south, N = north, TSS = transcription start site. **e** Differential analysis revealed up to 8 AD-related DMPs located within the HOXA genes cluster in the short arm of chromosome 7. The upper tracks show 450K microarray values and results of differential analysis. Blue dots represent per CpG median *β*-values for patients and controls. Vertical red bars represent *β*-difference values for CpGs included in the DMPs that crossed the statistical threshold (*β*-difference > 0.1 and *p* value < 0.05). Grey bars represent *β*-difference values for the CpGs included in the DMPs. Methylation values are aligned to ENCODE/Broad data for H3K27me3 histone marks in H1 human Embryonic Stem Cells (H1-hESC) at the bottom**. f** Dot plot graphs show 450K microarray *β*-values for the CpGs with most significant *p* value for each 4 genes within DMPs in the HOXA cluster. The selected CpGs are as follow, HOXA2: cg04027736, HOXA3: cg22962123, HOXA4:cg16651126, EVX1:cg08865099. **g** Extended mapping of hypermethylated DMPs within HOXA3 gene in 2 AD cases (below) compared to 2 controls (above) obtained by bisulfite cloning sequencing that shows how differential methylation affects multiple contiguous CpGs. The amplicon overlaps cg00921266 (blue arrow) and cg22962123 (red arrow). Black circles represent methylated cytosines while white circles denote unmethylated cytosines. Each column symbolizes a unique CpG site in the examined amplicon, and each line represents an individual DNA clone. Average percentage of methylation for each analyzed sample (control or patient) at this particular amplicon is indicated at the bottom of each sample

Differential methylation analysis between the AD and control hippocampus was performed by using the limma package (R/Bioconductor). After adjusting for age and false discovery rate (FDR) correction, the analysis revealed 118 AD-related differentially methylated positions (DMPs) (absolute *β*-difference ≥ 0.1 and adjusted *p* value ≤ 0.05) located next to 159 genes (Fig. 1, Table 1). Most of the DMPs, 102 (86.4%), were hypermethylated in AD cases compared to controls (Fig. 2a–c). Inspection of methylation patterns revealed that AD-related DMPs showed a mild-to-moderate effect size, as the average absolute *β*-difference was 0.12 (SD = 0.02) (Fig. 2b). These results are in line with previous AD methylome studies, which showed recurrent gains in DNA methylation of a mild-to-moderate effect size in other brain regions [9–14]. Indeed, up to 17 (10.7%) differentially methylated genes in our study are found among the top-ranked genes in previous AD methylome studies performed on frontal, temporal, or entorhinal cortex [11, 12, 14] (Additional file 1: Table S2).

**Table 1:** Differentially methylated positions (DMPs) in AD hippocampus measured by 450K Illumina BeadChip array

DMPs	Genomic coordinates		Beta. difference	FDR *p* value	GeneID1	GeneID2	Neurogenesis-related	Neurogenesis-related PMID	
cg02091185	5	170288766	0.198	0.003	RANBP17		No		
cg07333191	4	13526769	0.168	0.002	RAB28	NKX3-2	No		
cg14780466	2	20870812	0.150	0.011	GDF7	APOB	Yes	17293457	22897442
cg04370442	16	58019866	0.147	0.013	TEPP	ZNF319	No		
cg16867657	6	11044877	0.145	0.002	ELOVL2		No		
cg04154027	5	78985588	0.144	0.002	CMYA5		No		
cg19506623	2	161265259	0.143	0.036	RBMS1	ITGB6	No		
cg04498198	17	27899966	0.140	0.002	TP53I13	GIT1	Yes		25792865
cg23077820	2	223154176	0.140	0.002	PAX3	EPHA4	Yes	26287727	25978062
cg11015251	7	27170554	0.138	0.005	HOXA4		No		
cg16258854	2	20648194	0.138	0.005	RHOB	HS1BP3	Yes	15306568	
cg01463828	8	22446721	0.137	0.007	PDLIM2		No		
cg13935577	12	107974897	0.136	0.008	BTBD11	PWP1	No		
cg12100751	1	109203672	0.134	0.002	HENMT1		No		
cg19022697	1	55247140	0.133	0.004	PARS2	DHCR24	Yes	29410512	24842139
cg25840926	2	20647987	0.133	0.002	RHOB	HS1BP3	Yes	15306568	
cg22962123	7	27153605	0.131	0.008	HOXA3	HOXA2	Yes	12954718	10230789
cg05637536	1	154475068	0.130	0.002	TDRD10		No		
cg01331772	2	131094827	0.129	0.002	IMP4		No		
cg23279355	5	78985592	0.128	0.002	CMYA5		No		
cg01579024	5	170288757	0.127	0.013	RANBP17		No		
cg15548613	22	38610795	0.127	0.002	MAFF	TMEM184B	No		
cg13172549	7	27153636	0.125	0.019	HOXA3	HOXA2	Yes	12954718	10230789
cg02231404	20	62679635	0.124	0.008	SOX18		Yes	29666335	
cg13327545	10	22623548	0.124	0.002	SPAG6	BMI1	Yes	29666335	19212323
cg18247055	10	22634226	0.122	0.003	SPAG6		Yes	26130477	
cg00921266	7	27153663	0.122	0.039	HOXA3	HOXA2	Yes	12954718	10230789
cg09490371	2	233253024	0.120	0.011	ECEL1P2	ALPP	No		
cg17448336	3	147141588	0.119	0.047	ZIC1		Yes	17507568	
cg16127683	15	40268777	0.119	0.021	EIF2AK4	SRP14	No		
cg25774643	11	627175	0.119	0.028	SCT	CDHR5	Yes	21159798	
cg18121224	5	176559563	0.119	0.010	NSD1		No		
cg07816556	6	26017280	0.119	0.004	HIST1H4A	HIST1H3A	No		
cg16308533	17	40838983	0.118	0.004	CNTNAP1	EZH1	Yes	26740489	23932971
cg04533276	22	19709548	0.117	0.007	SEPT5	GP1BB	Yes	17935997	
cg22900415	13	20736075	0.117	0.010	GJA3		No		
cg22385702	2	45175881	0.116	0.025	SIX3	SIX2	Yes	17576749	11401394
cg09655403	5	78985495	0.116	0.004	CMYA5		No		
cg22507154	1	91185233	0.116	0.016	BARHL2		Yes	22307612	
cg24369989	15	78933807	0.115	0.002	CHRNB4		No		
cg16404157	14	38724648	0.115	0.010	CLEC14A		No		
cg03146625	12	54448729	0.115	0.018	HOXC4	SMUG1	No		
cg04027736	7	27143403	0.115	0.011	HOXA2		Yes	10230789	
cg12024906	19	37825679	0.115	0.005	HKR1		No		
cg00303378	1	159825552	0.115	0.002	VSIG8	C1orf204	No		
cg17508941	7	19183280	0.114	0.002	TWIST1	FERD3L	Yes	23555309	23254923
cg24079702	2	106015771	0.114	0.005	FHL2		No		
cg15084543	1	79472408	0.113	0.006	ELTD1		No		
cg25738176	17	3848506	0.113	0.033	ATP2A3	P2RX1	No		
cg20864214	11	73054121	0.113	0.018	RELT	ARHGEF17	No		
cg06452665	13	43148436	0.113	0.010	TNFSF11		Yes	24087792	
cg21572722	6	11044894	0.113	0.003	ELOVL2		No		
cg26092675	6	26225258	0.112	0.002	HIST1H3E		No		
cg07336350	16	54322127	0.111	0.013	IRX3		Yes	10704856	
cg07809484	19	51231968	0.111	0.002	GPR32	CLEC11A	No		
cg16651126	7	27170552	0.111	0.007	HOXA4		No		
cg05726109	22	19709755	0.111	0.007	SEPT5	GP1BB	Yes	17935997	
cg07584855	1	221055545	0.110	0.007	HLX	DUSP10	Yes	7907015	19139271
cg14566959	5	140772681	0.110	0.006	PCDHGA4		No		
cg02798280	19	39087135	0.110	0.007	MAP4K1	RYR1	No		17767953
cg24756378	14	33401638	0.110	0.027	NPAS3	AKAP6	Yes	21709683	
cg11864574	10	22635028	0.110	0.015	SPAG6		Yes	29666335	
cg06555959	8	61835620	0.110	0.050	CLVS1	CHD7	Yes		23827709
cg12253175	12	58132093	0.110	0.029	AGAP2		No		
cg15834355	12	54442075	0.110	0.014	HOXC4	HOXC5	Yes		23103965
cg09596958	12	58132105	0.109	0.031	AGAP2		No		
cg02267270	6	37616410	0.109	0.047	MDGA1	CCDC167	Yes	21104742	
cg18181229	1	164545699	0.109	0.016	PBX1	LMX1A	Yes	27226325	24172139
cg01421119	1	211555733	0.109	0.019	TRAF5	RD3	No		
cg03729251	4	151501035	0.108	0.012	LRBA	MAB21L2	Yes		11960703
cg22090150	17	4098227	0.107	0.002	ANKFY1	CYB5D2	No		
cg05877788	17	27899874	0.107	0.003	TP53I13	GIT1	Yes		25792865
cg06396119	13	49792767	0.106	0.025	MLNR		No		
cg22154659	7	27134369	0.106	0.015	HOXA1	SKAP2	Yes	14522873	
cg14266527	4	151501298	0.106	0.004	LRBA	MAB21L2	Yes		11960703
cg24177393	5	43037517	0.105	0.033	SEPP1	ZNF131	No		
cg07942135	7	27154262	0.105	0.022	HOXA3	HOXA2	Yes	12954718	10230789
cg22904711	19	44278628	0.105	0.041	KCNN4	SMG9	No		
cg01566965	4	174447847	0.104	0.028	HAND2	SCRG1	Yes	22323723	
cg14557699	5	140254909	0.104	0.002	PCDHA12		No		
cg26698460	19	58716004	0.104	0.010	ZNF274	ZNF544	No		
cg02287710	14	102027660	0.103	0.004	DIO3		Yes	27707971	
cg21415530	8	140715802	0.103	0.010	KCNK9		No		
cg07589899	2	62020677	0.103	0.002	XPO1	FAM161A	No		
cg20192747	18	44774846	0.103	0.003	SKOR2		Yes	24491816	
cg17179862	17	46681362	0.103	0.003	HOXB6	LOC404266	Yes	10686603	
cg26587870	6	27730563	0.103	0.010	ZNF184	HIST1H2BL	No		
cg01089914	2	218843229	0.102	0.017	RUFY4	TNS1	No		
cg14962509	1	36039655	0.102	0.004	TFAP2E		No		
cg21869609	19	2291613	0.102	0.018	LINGO3		Yes	18297755	
cg08865099	7	27281581	0.102	0.043	EVX1		Yes	10399918	
cg13836098	6	26225268	0.102	0.002	HIST1H3E		No		
cg00611789	5	78985432	0.102	0.011	CMYA5		No		
cg13771313	11	72533295	0.102	0.017	ATG16L2	FCHSD2	No		
cg21811021	4	6659346	0.101	0.015	S100P	MRFAP1	No		
cg11254700	19	53561386	0.101	0.020	ERVV-2	ZNF160	No		
cg14557202	12	54764371	0.101	0.028	ZNF385A	GPR84	No		
cg03422911	1	237205295	0.101	0.004	RYR2		Yes	17767953	
cg19153828	2	127782651	0.101	0.009	BIN1	GYPC	No		
cg06867571	11	65306934	0.101	0.019	LTBP3	SCYL1	No		
cg09317554	4	151505084	0.101	0.014	LRBA	MAB21L2	Yes		11960703
cg05404236	13	110437093	0.100	0.017	IRS2		Yes	28833887	
cg02771117	8	11279352	− 0.100	0.022	FAM167A	BLK	No		
cg10373891	13	52338758	− 0.101	0.017	DHRS12	WDFY2	No		
cg24607755	11	36171375	− 0.102	0.010	LDLRAD3	PRR5L	No		
cg20102280	13	47470793	− 0.102	0.004	HTR2A		No		
cg21735068	8	97975467	− 0.104	0.015	PGCP	TSPYL5	Yes		26911678
cg23907053	12	70215816	− 0.107	0.015	RAB3IP	MYRFL	No		
cg14701867	10	64193068	− 0.107	0.023	ZNF365	ADO	No		
cg14830371	8	25991602	− 0.108	0.027	PPP2R2A	EBF2	Yes		25762221
cg16668651	15	81316319	− 0.110	0.035	IL16	MESDC2	No		
cg06688910	8	122466955	− 0.110	0.027	SNTB1	HAS2	No		
cg07463059	1	158979810	− 0.119	0.010	IFI16		No		
cg05165025	14	74253312	− 0.119	0.046	PTGR2	ELMSAN1	No		
cg07677157	12	66050928	− 0.133	0.026	HMGA2	MSRB3	Yes	18640244	
cg11236550	1	167090757	− 0.138	0.012	DUSP27	POU2F1	No		18241856
cg20597486	1	158979841	− 0.151	0.002	IFI16		No		
cg19987768	17	750306	− 0.162	0.043	NXN	GLOD4	Yes	29037191	

The table shows 118 DMPs with *β*-difference > 0.100, prioritized by beta difference (delta) criteria. *FDR* false discovery rate, *Adj.* adjusted, *ID* identification, *PMID* PubMed identification. Each probe (CpG site) was annotated by UCSC hg19 build. The last two columns show the PMID of papers supporting the involvement of the genes into neurogenesis or neural development

Genomic distribution of AD-related DMPs was next analyzed. We observed that DMPs were more likely to locate in CpG islands, showing a 1.5-fold enrichment (*p* value < 0.001) compared with random expectation based on all probes included in the analysis. In addition, significant enrichment was found at first exon (2.4-fold, *p* value < 0.001) and body (1.2-fold, *p* value < 0.05) regions (Fig. 2d). Moreover, up to 13 (8.1%) genes were associated with 2 or more AD-related DMPs which would suggest the presence of hotspots of aberrant methylation gain in the human hippocampus affected by AD.

### Validation and additional mapping of DMPs

**Fig. 3:**
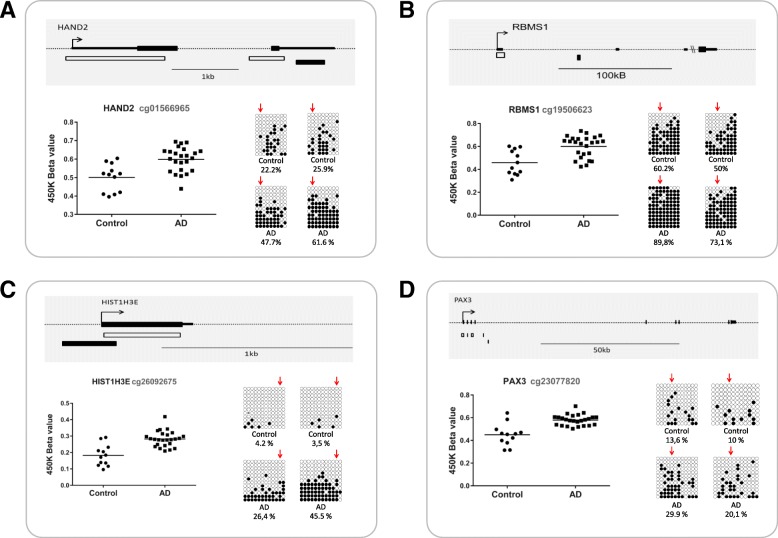
Validation and extended mapping for the differentially methylated genes *HAND2*, *RBMS1*, *HIST1H3E*, and *PAX3*. Bisulfite cloning sequencing experiments show that hypermethylation affects multiple contiguous CpGs located in the 3’UTR of *HAND2* (**a**), first exon of *RBMS1* (**b**), the promoter region of *HIST1H3E* (**c**), and the body of *PAX3* (**d**). The upper track of each panel shows a genomic map of each gene. White boxes below each gene denote CpG islands, and black boxes represent bisulfite cloning sequencing amplicons. Dot plot graphs show the results of the 450K array (beta values) for CpG probes. Validation results are represented by black/white circle-style figures. Each rectangle corresponds to one sample and shows the methylation pattern at a discrete genomic region surrounding the significant CpG probed by the 450K array which is marked by a red arrow. Black circles represent methylated cytosines while white circles denote unmethylated cytosines. Each column symbolizes a unique CpG site in the examined amplicon, and each line represents an individual DNA clone. Average percentage of methylation for each analyzed sample (control or patient) at this particular amplicon is indicated at the bottom of each sample

Among the 118 AD-related DMPs, we selected 7 DMPs to be validated based on their genomic location in or near genes relevant to brain function or AD pathology, including some of the candidate hotspots, i.e., *HAND2*, *HOXA3*, *HIST1H3E*, *NXN*, *PAX3*, *RBMS1*, and *RHOB*. All the selected genes were successfully validated by bisulfite cloning sequencing since a significant correlation was shown between the 450K array data and methylation levels obtained by bisulfite cloning sequencing (Additional file 1: Table S3). Additional mapping of the altered methylation pattern across multiple contiguous CpGs was generated (Fig. 2g and Fig. 3, Additional file 1: Figure S1). The results of this mapping support the idea that changes in DNA methylation are not confined to the CpGs queried by the array. On the contrary, alterations of DNA methylation in AD seem to be consistently distributed across discrete regions of the genome and to involve multiple contiguous CpGs.

### DNA methylation levels correlate with phosphorylated tau protein burden

The human hippocampus is particularly vulnerable to specific anatomopathological changes in AD and is considered the region where tau pathology initiates, together with the entorhinal cortex [17–19]. To explore whether the altered DNA methylation pattern reflects AD pathological changes in the hippocampus, we used a semi-automated quantitative method described in detail elsewhere [7] to measure the extension of phosphorylated tau (p-tau) deposits in our set of hippocampal samples. Next, Pearson’s coefficient was calculated to evaluate the correlation between DNA methylation levels at each of the identified 118 AD-related DMPs and the extension of p-tau deposits in the hippocampus. We found that DNA methylation levels in 43 (36.4%) DMPs were significantly correlated with the burden of p-tau deposits (Additional file 1: Table S4). The strongest correlation was observed for differentially methylated CpGs located close to the *SOX18*, *HKR1*, *PCDHA12*, and *ATG16L2* genes.

### AD-related DMPs overlap bivalent histone marks and poised promoters

Independent epigenetic mechanisms may play together to coordinately fine-tune gene expression. Therefore, we next asked whether the set of 118 AD-related DMPs was predicted to associate with other epigenetic features such as histone modifications. To this end, we first performed a functional in silico analysis for enrichment in histone marks using ENCODE/Broad data for human brain hippocampus, normal human astrocytes (NH-A), and H1 human Embryonic Stem Cells (H1hESC) identified through the WashU Epigenome Browser [22] and the UCSC Genome Browser [23].

**Fig. 4.**
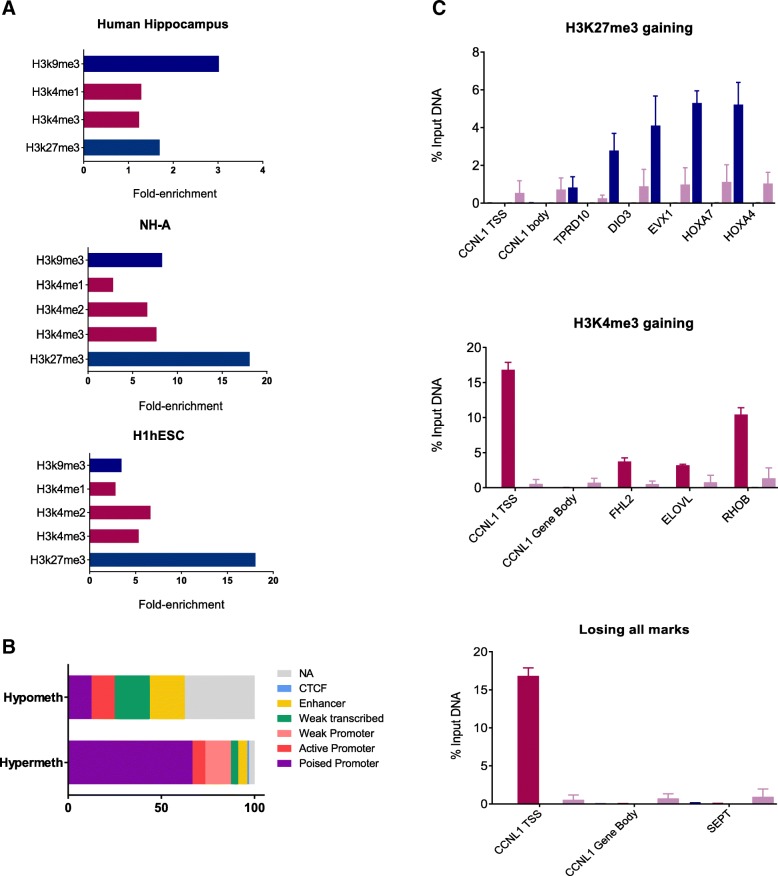
Histone marks enrichment and ChIP results in NHPCs. **a** The bar graph shows the strong enrichment in repressive histone marks (blue bars), particularly in H3K27me3 and H3K9me3, in our set of AD-related DMPs. Milder enrichment of active chromatin marks (red bars) is also shown. Only statistically significant results of the hypergeometric test for the available histone marks are shown. **b** DMPs that were hypermethylated occur preferentially in promoters in a poised or bivalent chromatin state. **c** A variety of fates for “poised” genes in committed NHPCs is represented by the results of ChIP experiments. Some of the promoters resolved to repressive (H3K27me3) or active (H3K4me3) promoters while others lost all histone marks. ChIP of the CCNL1 promoter and gene body regions were used as positive control for active promoter histone marks (H3K4me3) and negative control for repressive histone marks (H3K27me3)

We found that AD-related DMPs were overrepresented in regions of repressive histone marks, particularly in H3K27me3 mark in H1hESCs and H3K9me3 in the human hippocampus (Fig. 4a). We also realized that many of the AD-related DMPs shared both repressive (H3K27me3) and activating (H3K4me2 and H3K4me3) marks in the same locus. In other words, these DMPs overlapped regions of bivalent chromatin that usually characterizes poised promoters. Genes with poised promoters are generally repressed but ready for immediate activation in response to certain signals [24], and are thought to be key developmentally regulated genes not only in stem cells [24–27] but also in differentiated cells [27–29]. In our study, 70 (59.3%) AD-related DMPs overlapped poised promoters. When taking into account the direction of the methylation change, we observed that up to 68 (66.6%) of the hypermethylated DMPs overlapped bivalent promoters, whereas only 2 (12.5%) of the hypomethylated DMPs overlapped bivalent promoters (Fig. 4b).

### ChIP analysis of bivalent promoters in neural human progenitor cells

Although most of the bivalent modifications in hESC are usually resolved during lineage commitment, a small subset of poised promoters in hESC may remain bivalent during differentiation [24, 27]. Indeed, bivalent promoters may play complex roles in differentiated cells, keeping some genes poised for activation [24, 29, 30]. To explore the fate of bivalent chromatin modifications in our set of AD-related DMPs, we performed a number of chromatin immunoprecipitation-quantitative PCR (ChIP-qPCR) experiments in neural human progenitor cells (NHPCs) which represent a more differentiated state than hESC in the brain tissue. We ran ChIP-qPCR assays on NHPCs using anti-H3K27me3 and anti-H3K4me3 antibodies for selected differentially methylated genes, including the *HOXA* gene cluster. ChIP-qPCR analysis revealed that genes within the *HOXA* locus resolved to the repressive H3K27me3 status in NHPCs, as was also the case of the *DIO3* and *TDRD10* genes. On the contrary, *ROHB*, *ELOVL2*, and *FHL2* resolved to H3K4me3 active status in the committed NHPCs. Only *SEPT5* lost both H3K4me3 and H3K27me3 chromatin marks (Fig. 4c). These results illustrate a wide variety of histone marks fates for these differentially methylated genes which are poised in H1hESCs. Although additional research on this issue would be needed, these results suggest that AD-related DMPs are not enriched in genes that retain bivalent marks in lineage-committed cells.

### AD-related differentially methylated genes are linked to neural development and neurogenesis

Next, we wanted to know whether the set of AD-related DMPs was enriched for genes involved in specific diseases, functions, and pathways. To this end, we performed different levels of analysis. First, manually curated search using PubMed revealed that 50 (42.4%) AD-related DMPs were associated with genes related with neural development and neurogenesis (Table 1, Fig. 1). Consistently, most of these genes overlapped poised promoters (90.9%), since they are key developmentally regulated genes.

Next, we used the Genomic Regions Enrichment of Annotations Tool (GREAT) [31] to identify enriched ontological terms in our set of AD-related DMPs. The enriched gene ontology (GO) terms in the biological process category revealed a set of different processes consistently associated with embryonic and brain morphogenesis among others (Additional file 1: Figure S2). In addition, our set of AD-related DMPs was enriched in the “high mobility group (HMG) box domain binding” term in the GO molecular category (fold enrichment = 12.3; FDR *Q*-value < 0.01). HMG is a protein domain which confers proteins the ability to bind DNA and is related to a number of DNA processes, including transcription and DNA repair [32].

Next, an InterPro ontology analysis was performed to test for protein domains, families, and functional sites. The analysis showed enrichment for homeobox domain-related terms in the set of AD-related DMPs (Additional file 1: Table S5). Homeobox transcription factors are crucial in regulating pluripotency and cellular differentiation [33].

## Discussion

In this study, we profiled genome-wide DNA methylation levels to identify novel AD-related methylation changes in the human hippocampus. These results revealed genomic loci hypermethylated in AD cases compared to controls that largely overlap regulatory regions, mainly bivalent promoters. In addition, the DNA methylation signature was consistently related to genes crucial for neural development or neurogenesis and homeobox-containing transcription factors.

Neuropathological hallmarks of AD tend to occur in particularly vulnerable regions in the human brain [17, 20] and are associated to neuronal death and synapse loss from the very early stages of the disease [34]. However, molecular mechanisms underlying the specific brain region pattern of neuropathological changes in AD are not entirely clear. That was the rationale for selecting the human hippocampus to perform this epigenetic screening since it is a highly vulnerable region to AD neuropathological changes. In this regard, our analysis showed a statistically significant positive correlation between DNA methylation levels at 36.4% of the AD-related DMPs and the hippocampal burden of p-tau. On the other hand, and supporting the robustness of the present study, our results partially overlap those previously reported from methylome studies performed on other AD brain regions, such as prefrontal, frontal, and superior temporal neocortex or entorhinal cortex [9–14]. The overlap of DMPs among different AD brain regions suggests that a limited number of DMPs may be related to characteristic molecular processes of AD, regardless of the affected brain area. In any case, our complete set of AD-related DMPs extends and complements the current epigenomic landscape of the AD brain.

Interestingly, the functional analysis showed that a significant percentage of the differentially methylated genes were related to neural development and neurogenesis. It was astounding that other biological, cellular, and molecular processes generally associated with neurodegeneration such as apoptosis, autophagy, inflammation, oxidative stress, and mitochondrial or lysosomal dysfunction were not overrepresented in the set of AD-related DMPs. Though strongly related to brain development, neurogenesis is also maintained in the adult human brain, mainly in two distinct areas, i.e., the subventricular zone and the subgranular zone of the dentate gyrus in the hippocampus. There is substantial neurogenesis throughout life in the human hippocampus as it is estimated that up to one third of human hippocampal neurons are subject to constant turnover [35]. Adult neurogenesis is linked to hippocampal-dependent learning and memory tasks [36–38] and is reduced during aging [35, 39]. Recent evidence suggests that adult neurogenesis is altered in the neurodegenerative process of AD [40–42], but it is still controversial with some authors reporting increased neurogenesis [43, 44], whereas others show reduced neurogenesis [39, 42, 45, 46]. In the human hippocampus, a sharp drop in adult neurogenesis has been observed in subjects with AD [42]. Remarkably, protein tau has been also involved in the modulation of adult hippocampal neurogenesis exerted by external stimuli [47] and impairs proliferation of neuronal precursors in the hippocampal dentate gyros in a tauopathy mouse model [48]. Definitely, the molecular mechanisms involved in defective neurogenesis in AD remain to be elucidated [41].

In this scenario, the results of the present study point to neurogenesis-related genes as targets of epigenetic changes in the hippocampus affected by AD. Enrichment of AD-related DNA methylation marks in neurodevelopmental and neurogenesis-related genes may reflect changes in epigenetic regulation of the neuronal population subjected to exchange in the hippocampus, whose function and balance could be relevant to AD pathogenesis. These methylation changes might be built throughout life due to external and internal cues and would represent an example of epigenetic interaction between environmental and genetic factors in developing AD. As an alternative explanation, these epigenetic marks might also represent the trace of DNA methylation alterations induced during early developmental stages of the hippocampus, which would remain as a fingerprint in the larger proportion of hippocampal neurons that are not exchanged. This second hypothesis would link AD to early life stages, in concordance with recent studies that revealed abnormal p-tau deposits (pre-tangles) in brains of young individuals under 30 [49, 50] suggesting AD pathology would start earlier in life than it was previously thought. The influence of the genetic risk for AD has also been postulated to begin in early life [51], and other AD risk factors may be influenced by in utero environment [52].

We also observed that AD-related DMPs overlap relevant regulatory regions in the genome, such as CpG islands and bivalent histone marks corresponding to poised promoters. This result is in line with previous studies that found hypermethylated DMPs in the AD superior temporal gyrus to be enriched in poised promoters [14]. Promoters may be found in three distinct states: active, repressed, and poised. In the poised state, promoters are repressed but may be rapidly activated in response to certain cues [24]. Interestingly, poised promoters are very often associated with genes critical to the development and, as such, are characteristic of stem cells [24–27]. A number of poised promoters can also be maintained in lineage-committed and differentiated cells [29–31]. In our study, ChIP-qPCR analysis suggests that the bivalent promoters enriched in the set of AD-related DMPs resolve to a variety of states in committed NPCs and therefore are not enriched in genes retaining bivalent marks in lineage-committed cells.

The functional in silico analysis showed enrichment for the homeobox domain-containing family of genes in the set of AD-related DMPs. Homeobox domain-containing genes encode transcription factors (TFs) that result crucial during early embryonic development and morphogenesis [33]. Some homeobox TFs act by inducing cellular differentiation while other homeobox TFs are involved in maintaining pluripotency. Homeobox genes are known to be tightly regulated by DNA methylation and modifications of the chromatin state. Notably, homeobox domain-containing TFs are being closely connected to neurogenesis [53–55] and specific homeobox TFs, such as Dbx2, are involved in age-related neurogenic decline [56]. Most interestingly, Dbx2 is involved in the molecular changes that characterize the aged phenotype of neural stem/progenitor cells from the subventricular zone in mice, and it has been proposed as a player in promoting age-related neurogenic decline [56].

Deregulation of homeobox genes is related to certain diseases, such as cancer. However, the relationship with AD and neurodegenerative disorders has been barely assessed. Only a few reports are found in the literature regarding AD and homeobox domain-containing genes, e.g., low expression of *GAX* gene, a regulator of vascular differentiation, in brain endothelial cells in AD [57], or *GTX* gene, a homeobox gene with neuroprotective properties [58]. Therefore, these findings open a new avenue for research to better understand the role of homeobox TFs in the AD pathogenesis.

At any rate, we want to be cautious with our conclusions. There was a significant difference in age between controls and AD patients, being the latter group older than the former group. Although we adjusted for age in the statistical differential methylation analysis, the accuracy of this correction may be limited as there is little overlap in the age ranges of both groups. For the sake of external validity, these findings should be replicated in an independent cohort of human hippocampal samples.

## Conclusions

On the whole, our results suggest that altered DNA methylation in the AD hippocampus occurs at specific regulatory regions that are crucial for neural differentiation and support the notion that adult hippocampal neurogenesis may play a role in the development of AD. However, we are far from understanding how DNA methylation changes, interacting with other epigenetic mechanisms, modulate relevant molecular pathways in developing the disease. In addition, other topics such as the role of non-CpG methylation or hydroxymethylation would be interesting to address in the AD hippocampus. Therefore, further research on the alterations of epigenetic mechanisms in AD is guaranteed.

## Methods

### Aim, design, and setting of the study

The aim of this study was to profile genome-wide DNA methylation in the human hippocampus, a brain region particularly vulnerable to AD and the core of pathological protein tau deposits. This is an observational, transversal, case-control study to identify differentially methylated positions among AD cases and controls.

### Human brain samples and neuropathological examination

We evaluated postmortem hippocampal samples from 38 subjects (26 AD patients and 12 controls), provided by the Navarrabiomed Brain Bank. After death, half brain specimens from donors were cryopreserved at − 80 °C. Neuropathological examination was performed following the usual recommendations [59]. Assessment of β-amyloid deposit was carried out by immunohistochemical staining of paraffin-embedded sections (3–5 μm thick) with a mouse monoclonal (S6F/3D) anti-β-amyloid antibody (dilution 1/50) (Leica Biosystems Newcastle Ltd, Newcastle upon Tyne, UK). Evaluation of neurofibrillary pathology was performed with a mouse monoclonal antibody anti-human PHF-TAU, clone AT-8 (Tau AT8) (dilution 1/1000) (Innogenetics, Gent, Belgium), which identifies p-tau [19]. The reaction product was visualized using an automated slide immunostainer (Leica Bond Max) with Bond Polymer Refine Detection (Leica Biosystems Newcastle Ltd). AD staging was performed by using the ABC score according to the updated National Institute on Aging-Alzheimer’s Association guidelines [60]. Agreement for any diagnosis was reached by members of a panel composed of two neuropathologists (VZ, CE) and two neurologists (JS, MM).

### Genome-wide DNA methylation profiling and differential methylation analysis

CpG methylation levels were profiled genome-wide by using Infinium HumanMethylation450 BeadChip array (Illumina, Inc., San Diego, CA, USA) [61] at the Roswell Park Cancer Institute Genomics Shared Resource (Buffalo, NY, USA). Briefly, 500 ng of genomic DNA from each brain sample was bisulfite treated and hybridized to the BeadChip according to the manufacturer’s protocol. A total of 485,577 cytosine positions were interrogated throughout the human genome, covering the 99% of RefSeq genes and 96% of CpG islands.

### Quality control and data processing

In order to minimize the potential bias introduced by batch effects, we performed samples-to-batch allocation using the OSAT tool [62]. Microarray image processing was carried out using Genome Studio Methylation Module (v1.8.5). Background was corrected, and adjustment was performed to avoid type I/II assay chemistry bias. So as to minimize technical variation and improve data quality, the Dasen method [63] was used as a normalization tool.

Before performing differential methylation analysis, we removed probes that overlapped common single nucleotide polymorphisms (SNPs) and also those probes classified as internal controls of the Illumina microarray. Additionally, probes located on the X and Y chromosomes were discarded along with those probes previously described to hybridize to multiple locations in the genome [64, 65]. Probes that technically did not pass the Illumina quality threshold (1188 probes with bead count < 3 in > 5% of samples and 378 probes having 1% of samples with a detection *p* value > 0.05) were also removed. In the end, a total of 264,031 probes (representing individual CpG sites) were further analyzed for differential methylation (Additional file 1: Figure S3).

### Differential methylation analysis

Our aim was to identify differentially methylated positions (DMPs), that is to say, differentially methylated CpGs related with AD status. Linear model of microarray analysis (LIMMA) adjusted for age was performed to fit a linear regression model for each CpG site (R/Bioconductor package) [66]. Percentage of methylation (*β*-value) at each surveyed CpG site was calculated and ranged from 0 to 1. Benjamini and Hochberg false discovery rate (FDR) correction was used (*p* value < 0.05). Methylation differences were prioritized by lowest adjusted *p* values to ensure the most consistent DMPs between AD patients and controls. This analysis identified sets of candidate loci with consistent differences in methylation in AD versus control hippocampus. Gene annotation was obtained using the Genomic Regions Enrichment of Annotations Tool (GREAT) [31].

### Bisulfite sequencing validation of DMPs

Next, 500 ng of genomic DNA was bisulfite converted using the EpiTect Bisulfite Kit (QIAGEN, Redwood City, CA, USA) according to the manufacturer’s instructions. Primer pair sequences were designed by MethPrimer [67] and are listed in Additional file 1: Table S6. PCR products were cloned using the TopoTA Cloning System (Invitrogen, Carlsbad, CA, USA), and between 12 and 24 independent clones were sequenced for each examined subject and region by Sanger sequencing [68]. Methylation graphs were obtained by using QUMA software [69], and maps of genes were drawn by using IGV software.

### Quantitative assessment of p-tau deposits in the hippocampus

In order to quantitatively assess p-tau burden in the hippocampal samples of AD subjects, we applied a method described in detail elsewhere [7]. Briefly, sections of the hippocampus were examined after performing immunostaining with anti-p-tau antibody (clone AT-8) (dilution 1/1000), and representative images were analyzed with ImageJ software to obtain an average quantitative measure of the global p-tau deposit for each section and patient. Examples of AT-8 staining for control and different AD stages are shown in Additional file 1: Figure S4.

### Chromatin immunoprecipitation in Normal Human Neural Progenitor Cells

Normal Human Neural Progenitor (NHNP) cells (Lonza) were grown in 75 cm^2^ culture flasks in NPBM medium (Neural Progenitor Basal Medium, Lonza) with the addition of hFGF, hEGF, NSF-1, and GA. NHNP cells were fixed in 1% formaldehyde for 10 min at room temperature; the reaction was stopped by addition of 1.25 M glycine solution, and cells were washed in PBS and harvested in IP buffer (1 volume of SDS buffer to 0.5 volume of Triton dilution buffer and protease inhibitors).

For each ChIP, 100 μg of DNA was used. Chromatin was sonicated to an average size of 750 bp. Sonicated sample was then blocked by incubating with Protein G and A sepharose beads at 4 °C for 1 h. Ten percent of the sample was kept aside as INPUT, and 1 μg of antibody or IgG was added to the remaining sample and incubated overnight at 4 °C. The next day Protein G and A sepharose beads were added and incubated for 2 h at 4 °C. After extensive washes, immunocomplexes were eluted from the beads and cross-links were reversed. The DNA was recovered by phenol-chloroform extraction and ethanol precipitation. DNA was resuspended in 150 μl of water, and 3.75 μl were used for real-time qPCRs in a final volume of 10 μl. The antibodies used in this study were anti-H3 (ab1791, Abcam), anti-H3K27me3 (ab6002, Abcam), and anti-H3K4me3 (ab8580, Abcam). ChIP of the *CCNL1* promoter and gene body regions were used as a positive control for active promoter histone marks (H3K4me3) and negative control for repressive histone marks (H3K27me3).

### Functional in silico analysis of DMPs

We performed a systematic manual curation of the literature using PubMed to identify whether AD-related differentially methylated genes were enriched in nervous system functions, including neurogenesis and neural development.

In order to determine the biological significance of AD-related DMPs, gene ontology analysis and pathway analysis were performed using the Genomic Regions Enrichment of Annotations Tool (GREAT) [31]. To define gene regulatory domains, each gene was assigned a basal regulatory domain of a minimum distance upstream (5.0 kb) and downstream of the transcription start site (TSS) (1 Kb plus distal up to 1000 Kb). The gene regulatory domain was extended in both directions to the nearest gene’s basal domain but no more than the maximum extension in one direction. By using GREAT, we got InterPro ontology which contains data on protein domains, families, and functional sites. InterPro annotations give information about the function, structure, and evolution of the domains by combining several other databases (PROSITE, PRINTS, Pfam, ProDom, SMART, TIGRFAMs, PIRSF, SUPERFAMILY, PANTHER, and Gene3D). Only those terms with a FDR-corrected *p* value less than 0.05 were reported.

## Additional file

Additional file 1: **Table S1.** Brain sample set analyzed by 450 K Illumina BeadChip array. **Table S2.** Differentially methylated genes in previous AD methylome studies. **Table S3.** Correlation between 450K array data and DNA methylation levels obtained by bisulfite cloning sequencing. **Table S4.** Correlation between DNA methylation levels at each DMPs and tau burden. **Table S5.** InterPro Gene Ontology enrichment analysis. **Table S6.** Bisulfite PCR primers. **Figure S1.** Validation and extended mapping for the differentially methylated genes *RHOB* and *NXN*. **Figure S2.** Functional in silico study of DMPs. **Figure S3.** Bioinformatics pipeline. **Figure S4.** Representative examples of tau staining (AT8) for control and AD stages. (PDF 886 kb)

## Abbreviations

AD: Alzheimer’s disease
bp: Base pair
ChIP: Chromatin immunoprecipitation
CpG: Cytosine-phosphate-guanine dinucleotide
DMPs: Differentially methylated positions
GO: Gene ontology
GREAT: Genomic Regions Enrichment of Annotations Tool
GWAS: Genome-wide association studies
H1hESC: H1 human Embryonic Stem Cells
NH-A: Normal human astrocytes
NHNP: Normal Human Neural Progenitor Cells
NPBM: Neural Progenitor Basal Medium
OSAT: Optimal Sample Assignment Tool
PMI: Postmortem interval
p-tau: Hyperphosphorylated tau
SD: Standard deviation
SNPs: Single nucleotide polymorphisms
STRING: Search Tool for the Retrieval of Interacting Genes
TF: Transcription factor
TSS: Transcription start site
UCSC: University of California, Santa Cruz
