# Supplementary material for: DNA methylation signature of human hippocampus in Alzheimer’s disease is linked to neurogenesis
Source: Clin Epigenetics. 2019 Jun 19;11:91. doi: 10.1186/s13148-019-0672-7 (PMC6585076; doi:10.1186/s13148-019-0672-7)
Supplement: Supplementary file 1 — Table S1. Brain sample set analyzed by 450 K Illumina BeadChip array. Table S2. Differentially methylated genes in previous AD methylome studies. Table S3. Correlation between 450K array data and DNA methylation levels obtained by bisulfite cloning sequencing. Table S4. Correlation between DNA methylation levels at each DMPs and tau burden. Table S5. InterPro Gene Ontology enrichment analysis. Table S6. Bisulfite PCR primers. Figure S1. Validation and extended mapping for the differentially methylated genes RHOB and NXN. Figure S2. Functional in silico study of DMPs. Figure S3. Bioinformatics pipeline. Figure S4. Representative examples of tau staining (AT8) for control and AD stages. (PDF 886 kb) [file 13148_2019_672_MOESM1_ESM.pdf]

Supplemental Table S1. Brain sample set analyzed by 450K Illumina BeadChip array.

No.	Status	ABC score	Braak & Braak stage	APS	Age at death (years)	Gender	PMI (h)	Region hippocampus
1	Control	control	NA	NPD	61	male	8	yes
2	Control	control	NA	NPD	81	male	10.5	yes
3	Control	control	NA	NPD	43	female	3	yes
4	Control	control	NA	NPD	88	male	9	yes
5	Control	control	NA	NPD	53	male	7	yes
6	Control	control	NA	NPD	41	male	3.5	yes
7	Control	control	NA	NPD	28	male	6	yes
8	Control	control	NA	NPD	46	female	7	yes
9	Control	control	NA	NPD	69	male	12	yes
10	Control	control	NA	NPD	19	female	NA	yes
11	Control	control	NA	NPD	26	male	6.2	yes
12	Control	control	NA	NPD	54	male	18	yes
13	AD	A1B1C1	I	0.00	60	male	15.3	yes
14	AD	A1B1C1	I	2.00	85	male	3.2	yes
15	AD	A1B1C1	II	0.00	66	female	1.4	yes
16	AD	A1B2C1	III	0.00	88	female	33	yes
17	AD	A1B2C1	III	3.00	96	female	1.5	yes
18	AD	A1B2C2	III	0.33	79	female	13	yes
19	AD	A1B2C2	III	1.00	81	female	9	yes
20	AD	A1B2C3	IV	2.33	84	female	13	yes
21	AD	A2B2C2	III	5.00	88	male	3.5	yes
22	AD	A2B2C2	IV	2.33	91	female	10	yes
23	AD	A2B2C2	IV	1.33	84	male	3.3	yes
24	AD	A2B2C2	IV	3.00	97	female	NA	yes
25	AD	A2B2C3	IV	1.33	78	male	5	yes
26	AD	A2B3C3	V	5.67	77	female	4	yes
27	AD	A3B2C1	IV	1.33	90	female	3	yes
28	AD	A3B2C3	III	6.67	98	female	3	yes
29	AD	A3B2C3	III	2.67	85	female	NA	yes
30	AD	A3B3C2	V	3.00	92	female	14	yes
31	AD	A3B3C2	V	7.00	82	female	9	yes
32	AD	A3B3C2	V	8.00	91	male	5	yes
33	AD	A3B3C3	V	4.00	77	female	11	yes
34	AD	A3B3C3	VI	3.33	93	female	3	yes
35	AD	A3B3C3	VI	4.33	61	male	10	yes
36	AD	A3B3C3	VI	9.67	70	male	2.35	yes
37	AD	A3B3C3	VI	8.33	59	male	4	yes
38	AD	A3B3C3	VI	8.33	59	male	4	yes

The table shows the characteristic of the samples included in the study. No.: Number; AD: Alzheimer's disease; APS: amyloid plaque score; NPD: no protein deposit; PMI: post mortem interval; h: hours; NA: not applicable.

Supplemental Table S2. Differentially methylated genes in previous AD methylome studies.

Genes	AD methylome study
ATG16L2	De Jager et al.
BIN1	De Jager et al.; Yu et al.
CMYA5	Watson et al.; Bakulski et al.
DUSP27	Lunnon el al.
GP1BB	Lunnon el al.
HOTAIRM1	De Jager et al.
HOXA1	De Jager et al.
HOXA2	Lunnon el al.; De Jager et al.
HOXA3	Lunnon el al.
HOXA4	De Jager et al.
KCNN4	De Jager et al.
MAP4K1	Lunnon el al.
NXN	De Jager et al.
PARS2	De Jager et al.
SEPT5	Lunnon el al.
SIX3	De Jager et al.
SMG9	De Jager et al.

The table shows the genes that have been previously found associated with AD in methylome studies performed on human brain samples.

Supplemental Table S3. Correlation between 450K array data and DNA methylation levels obtained by bisulfite cloning sequencing

Gene	Array Probes	Pearson's Coefficient	p-value
HOXA3	cg22962123, cg13172549, cg00921266	0.784	0.002
HAND2	cg01566965, cg1967399	0.855	0.007
RBMS1	cg19506623, cg03157115, cg18852574	0.777	0.003
HIST1H3E	cg26092675, cg13836098	0.847	0.008
PAX3	cg23077820, cg04688351, cg22989843	0.934	<0.0001
NXN	cg19987768	0.982	0.017
RHOB	cg16258854	0.986	0.013

Supplemental Table S4. Correlation between DNA methylation levels at each DMPs and tau burden.

Probe ID	Genomic coordinates		GeneID1	GeneID2	Pearson's correlation coefficient	p-value	FDR corrected p-value
cg12253175	12	58132093	*AGAP2*		0.350*	0.037	0.045
cg09596958	12	58132105	*AGAP2*		0.370*	0.026	0.045
cg22090150	17	4098227	*ANKFY1*	*CYB5D2*	0.404*	0.015	0.042
cg13771313	11	72533295	*ATG16L2*	*FCHSD2*	0.436**	0.008	0.042
cg19153828	2	127782651	*BIN1*	*GYPC*	0.353*	0.035	0.045
cg13935577	12	107974897	*BTBD11*	*PWP1*	0.389*	0.019	0.042
cg24369989	15	78933807	*CHRNB4*		0.407*	0.014	0.042
cg04154027	5	78985588	*CMYA5*		0.406*	0.014	0.042
cg23279355	5	78985592	*CMYA5*		0.348*	0.038	0.045
cg00611789	5	78985432	*CMYA5*		0.350*	0.037	0.045
cg09490371	2	233253024	*ECEL1P2*	*ALPP*	0.349*	0.037	0.045
cg16127683	15	40268777	*EIF2AK4*	*SRP14*	0.414*	0.012	0.042
cg13836098	6	26225268	*HIST1H3E*		0.392*	0.018	0.042
cg12024906	19	37825679	*HKR1*		0.517**	0.001	0.020
cg07584855	1	221055545	*HLX*	*DUSP10*	0.379*	0.023	0.045
cg22962123	7	27153605	*HOXA3*	*HOXA2*	0.349*	0.037	0.045
cg13172549	7	27153636	*HOXA3*	*HOXA2*	0.400*	0.016	0.042
cg00921266	7	27153663	*HOXA3*	*HOXA2*	0.355*	0.034	0.045
cg17179862	17	46681362	*HOXB6*	*LOC404266*	0.344*	0.040	0.045
cg20597486	1	158979841	*IFI16*		-0.397*	0.017	0.042
cg01331772	2	131094827	*IMP4*		0.335*	0.046	0.048
cg09317554	4	151505084	*LRBA*	*MAB21L2*	0.346*	0.039	0.045

cg02798280	19	39087135	*MAP4K1*	*RYR1*	0.394*	0.018	0.042
cg02267270	6	37616410	*MDGA1*	*CCDC167*	0.373*	0.025	0.045
cg06396119	13	49792767	*MLNR*		0.422*	0.010	0.042
cg24756378	14	33401638	*NPAS3*	*AKAP6*	0.378*	0.023	0.045
cg19022697	1	55247140	*PARS2*	*DHCR24*	0.409*	0.013	0.042
cg14557699	5	140254909	*PCDHA12*		0.444**	0.007	0.042
cg01463828	8	22446721	*PDLIM2*		0.360*	0.031	0.045
cg20864214	11	73054121	*RELT*	*ARHGEF17*	0.418*	0.011	0.042
cg25840926	2	20647987	*RHOB*	*HS1BP3*	0.404*	0.015	0.042
cg03422911	1	237205295	*RYR2*		0.340*	0.043	0.047
cg21811021	4	6659346	*S100P*	*MRFAP1*	0.330*	0.049	0.049
cg05726109	22	19709755	*SEPT5*	*GP1BB*	0.335*	0.046	0.048
cg22385702	2	45175881	*SIX3*	*SIX2*	0.330*	0.049	0.049
cg02231404	20	62679635	*SOX18*		0.550**	0.001	0.020
cg05637536	1	154475068	*TDRD10*		0.362*	0.030	0.045
cg14962509	1	36039655	*TFAP2E*		0.354*	0.034	0.045
cg04498198	17	27899966	*TP53I13*	*GIT1*	0.357*	0.032	0.045
cg05877788	17	27899874	*TP53I13*	*GIT1*	0.399*	0.016	0.042
cg01421119	1	211555733	*TRAF5*	*RD3*	0.403*	0.015	0.042
cg00303378	1	159825552	*VSIG8*	*C1orf204*	0.359*	0.031	0.045
cg07589899	2	62020677	*XPO1*	*FAM161A*	0.345*	0.039	0.045

ID: identification; Genomic coordinates according to GRCh37/hg19 Assembly; GeneID1 & GeneID2: gene aliases of overlapping genes; * p-value<0.05; ** p-value<0.01

Supplemental Table S5. InterPro Gene Ontology enrichment analysis.

Ontology	# Term Name	Hyper Rank	Hyper FDR Q-Val	Hyper Fold Enrichment	Hyper Foreground Region Hits	Hyper Total Regions	Hyper Region Set Coverage	Hyper Foreground Gene Hits	Total Genes Annotated
InterPro	Homeodomain-like	4	1.34044e-4	3.6792	23	14059	19.49%	21	327
	Homeobox domain	5	2.95230e-4	3.9476	20	11394	16.95%	18	243
	Homeobox, conserved site	3	3.24527e-5	4.6678	20	9636	16.95%	17	188
	Homeodomain, metazoa	2	2.74152e-5	6.8887	15	4897	12.71%	11	92
	B30.2/SPRY domain	10	2.48293e-2	10.5090	6	1284	5.08%	3	91
	SPla/RYanodine receptor SPRY	9	2.52847e-2	10.6753	6	1264	5.08%	3	89
	Homeobox protein, antennapedia type	7	6.26551e-3	21.0968	5	533	4.24%	4	12
	Homeobox protein, antennapedia type, conserved site	1	1.01380e-8	32.3586	10	695	8.47%	6	20

	Domain of unknown function DUF4074	6	7.82430e-4	65.6620	4	137	3.39%	1	3
	Mab-21-like	8	1.31348e-2	71.7742	3	94	2.54%	1	2
	HIN-200/IF120x	11	2.55725e-2	249.8804	2	18	1.69%	1	4

The table shows the top-ranked categories obtained by using the InterPro Ontology set.

Supplemental Table S6. Bisulfite PCR primers.

Gene ID	Probe	Amplicon size	Tm1	Forward Primer	Tm2	Reverse Primer	CpGs in amplicon
HAND2	cg01566965	397	56.59	TTTTTTTGAGGTATTAGTTATTAAAAGATT	56.36	TCCCTCTTAACTATATAAACACCAAC	9
HIST1H3E	cg13836098	279	57.65	GAGTTGTTTTAGTGGTTAGTTGTTTG	59.94	AAAAAAACCAATTCTCTATCCAAATTTA	12
HOXA3	cg00921266	327	54.30	AGTAAGAGAGTTTTTTTGTGAGAGT	50.30	AACTCTACCTAAACTAATAACACC	20
NXN	cg19987768	263	50.72	GTTTTAAATGTTATTATAAATTTTAAGT	58.26	ATTCTACCAAAAAACAAAACTTTCC	12
PAX3	cg23077820	190	57.58	GGGTTTTTATTGAGTAATAATTATTGAAG	52.99	ACATTCATAAATACTAAAACCATTC	12
RBMS1	cg19506623	179	59.62	ATAAAGGGAGGAGGGATTTTTTT	58.37	TCCCAATAACTTATCCAAAAACC	9
RHOB	cg16258854	216	59.73	TTGGGTTTTTTATTGAGTGTTAAGG	57.76	TACAAACAAAAAATATCAAACTCCC	12

ID: identification; Tm1: Melting Temperature Forward Primer; Tm2: Melting Temperature Reverse Primer

Supplemental Figure S1

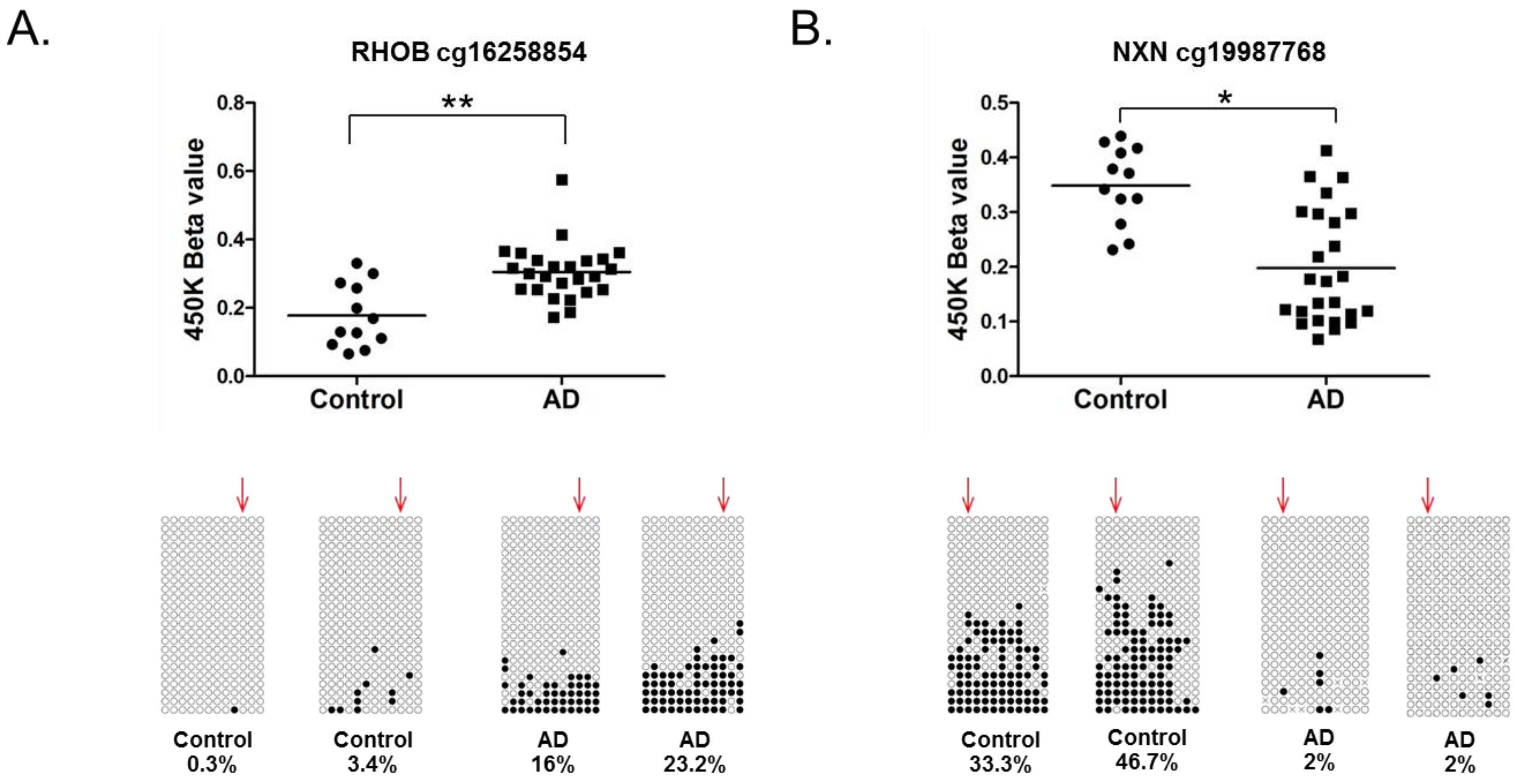

Validation and extended mapping for the differentially methylated genes *RHOB* and *NXN*. Bisulfite cloning sequencing shows that hypermethylation affects multiple contiguous CpGs within *RHOB* gene (A) and hypomethylation affects multiple contiguous CpGs within *NXN* gene (B). White boxes below each gene denote CpG islands and black boxes represent bisulfite cloning sequencing amplicons. Dot-plot graphs show the results of the 450K array (beta values) for CpG probes. Validation results are represented by black/white circle-style figures. Each rectangle corresponds to one sample and shows the methylation pattern at a discrete genomic region surrounding the significant CpG probed by the 450K array which is denoted by a red arrow. Black circles represent methylated cytosines while white circles denote unmethylated cytosines. Each column symbolizes a unique CpG site in the examined amplicon and each line represents an individual DNA clone. Average percentage of methylation for each analyzed sample (control or patient) at this particular amplicon is indicated at the bottom of each sample.

Supplemental Figure S2

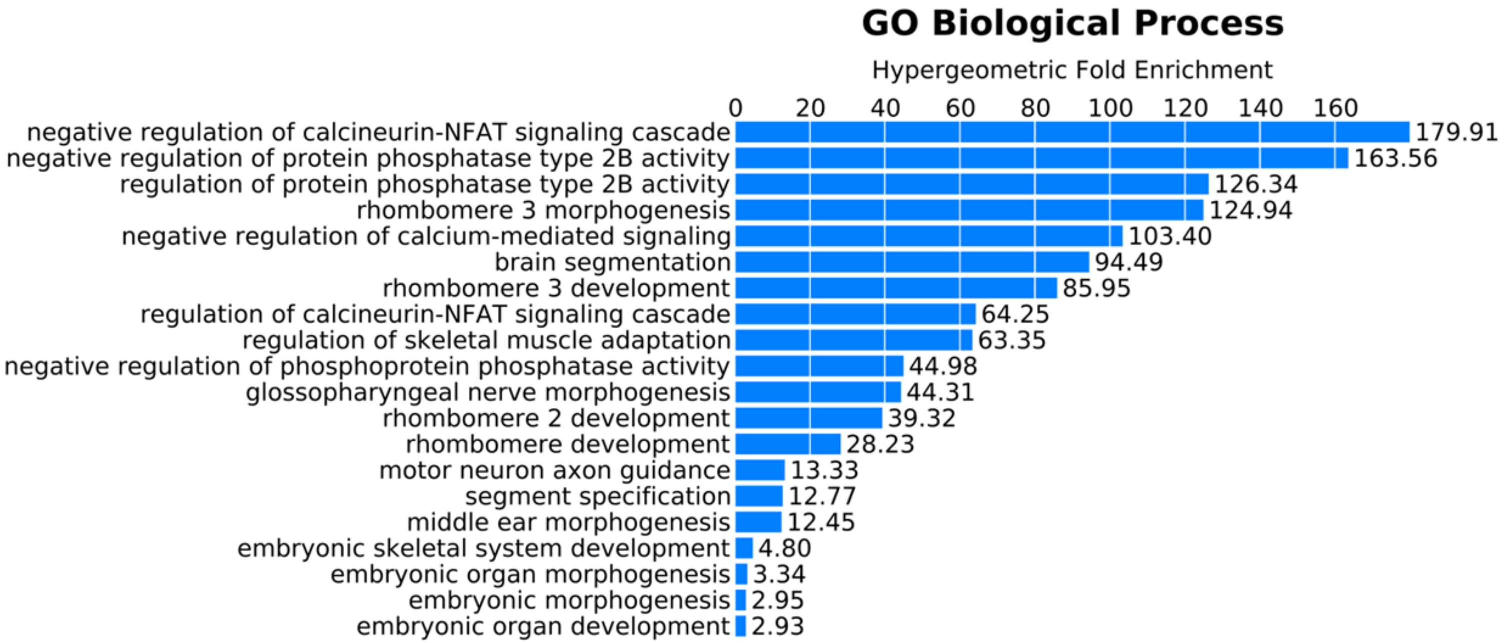

Functional *in silico* study of DMPs. The bar graph shows the results of the InterPro ontology analysis that revealed strong enrichment in processes consistently associated with embryonic and brain morphogenesis among others in our set of DMPs in the hippocampus of AD patients.

Supplemental Figure S3

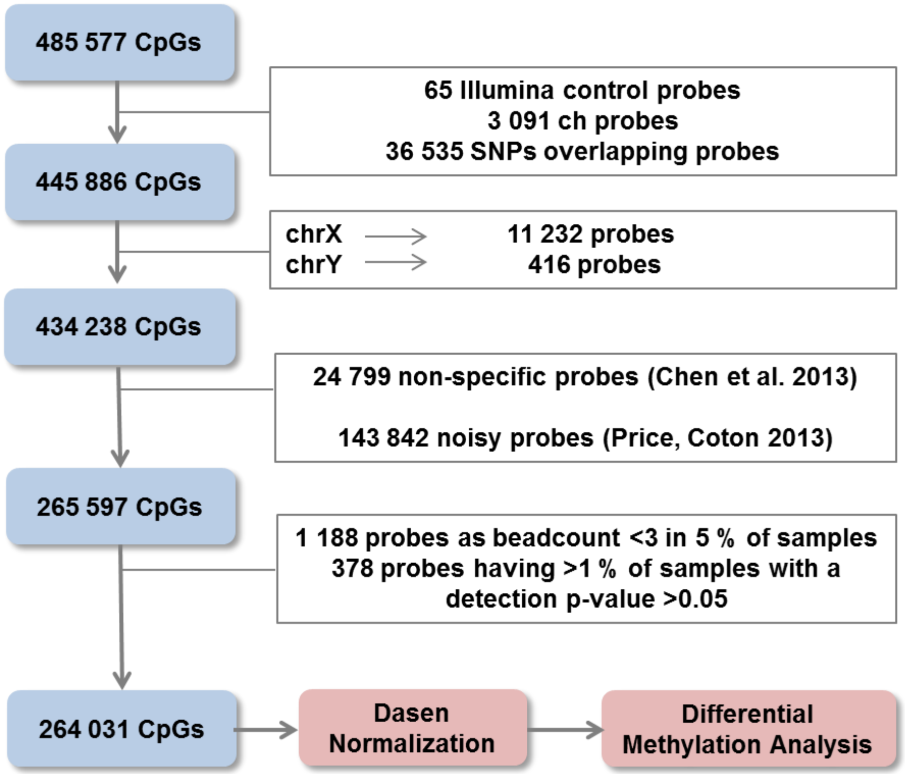

The diagram shows the bioinformatics pipeline used in this study: procedures for 450K methylation data quality control and normalization analysis.

Supplemental Figure S4

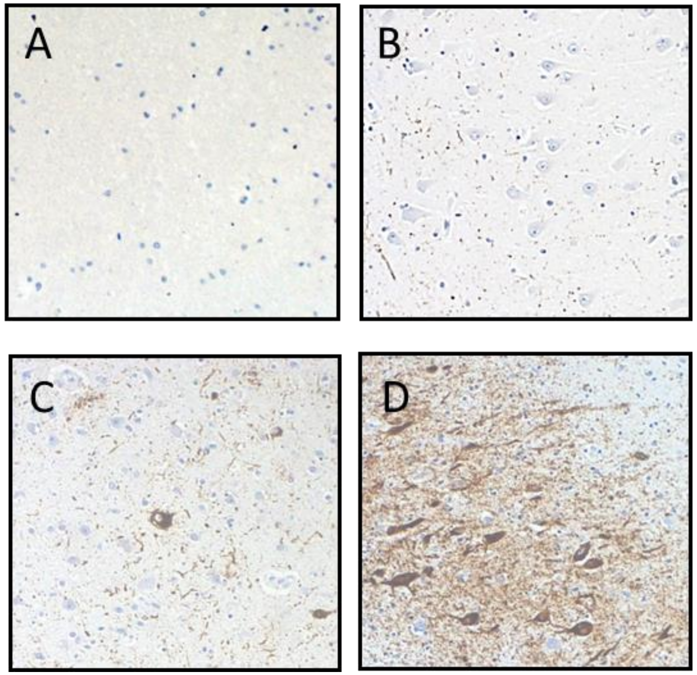

Representative examples of tau staining (AT8) for control and AD stages. Pictures were obtained at 10 x from cases showing different degree of protein tau deposit. A-D: AT-8 staining. Density degree of neurophil threads and tangles (A, control; B, low; C, intermediate; D, high).
